# Assessing the Plain Language Planner for Communication About Common Palliative Care Medications

**Published:** 2017-09-01

**Authors:** Elaine Wittenberg, Betty Ferrell, Joy Goldsmith

**Affiliations:** 1 Communication Studies, California State University, Los Angeles, California;; 2 City of Hope National Medical Center, Division of Nursing Research and Education, Duarte, California;; 3 Department of Communication, University of Memphis, Memphis, Tennessee

## Abstract

Using plain language to communicate about oncology and palliative care medications and symptoms is recommended as a communication strategy to address patient/family health literacy demands. This study tested the Plain Language Planner©, a provider tool for communicating about medication and symptoms using plain language. Prior to and immediately following an oncology and palliative care nurse educational session, participants (n = 87) role-played about a symptom and medication. Common symptoms (nausea, constipation, and anxiety) and medications were selected. Self-evaluation and peer evaluation addressing the extent of plain language used during the role-play were rated. Plain language characteristics improved post educational role-play sessions for nurses. The largest improvement in plain language was the inclusion of the brand and generic names of medication in relating the drug to the symptom. The pocket guide provided during the educational session was consulted by 86% of nurses during the postsession role-play. Brief training with the Plain Language Planner may improve provider communication and meet patient/family health literacy needs. This resource may be a valuable asset to other health-care disciplines working in oncology and palliative care contexts.

Clear communication about pain medication and symptoms is necessary to ensure oncology patients and family caregivers understand the purpose of the medication, can anticipate side effects, and are able to administer medication on their own. Patient-centered pain management addresses the different knowledge bases and skills between staff and patient and involves communicating pain management goals to both patient and family (Hayes & Gordon, 2015). Health organizations including the National Institutes of Health and the American Medical Association have recommended that the level of educational health information should be no higher than a sixth-grade reading level, whereas the Centers for Disease Control and Prevention recommend that reading levels be lower than eighth-grade readability (Badarudeen & Sabharwal, 2010). Patients with active communication about pain, including asking questions and giving information, have been reported to have better pain relief (Smith, DuHamel, Egert, & Winkel, 2010).

Using plain language when communicating with patients and families has been recommended as an important component of provider education and training to address health literacy demands and disparities in care (Nouri & Rudd, 2015). In short, plain language calls on the provider to speak/write with less jargon, use an active voice, and communicate in shorter sentences (Centers for Disease Control and Prevention, 2016). One way to accomplish better communication is through the teach-back method, which evaluates patient understanding by asking a patient to restate and explain the information relayed by the health-care provider. This method also provides clinicians with a better way to determine when information becomes distressing to the patient or when to stop offering information (Chou, Gaysynsky, & Persoskie, 2015).

Although the teach-back method is a common intervention strategy for reducing health literacy barriers between provider and patient/family, it is a generalized approach that does not address the specific nuances of communication about pain medication and symptoms. In one study aimed at teaching residents to use plain language, self-assessment reports showed high perceived self-efficacy in using the teach-back method; however, actual observed interactions revealed high use of jargon and low efficacy of the technique (Howard, Jacobson, & Kripalani, 2013).

## BACKGROUND ON THE PLAIN LANGUAGE TOOL

The Plain Language Planner for Palliative Care© (PLP) is a tool for communicating about medications and symptoms in plain language at the sixth-grade level. Using the essential medications identified by the World Health Organization for treatment of common palliative symptoms, the PLP was developed as part of the curriculum for a National Cancer Institute–funded national nurse communication training program called COMFORT (Wittenberg-Lyles, Goldsmith, Ferrell, & Ragan, 2012). The PLP translates common medications and symptoms in palliative care into plain language.

Previously, we set out to explore how the PLP would influence providers’ explanation of a medication to address a patient symptom. A comparison of written responses before and after an education session showed improvement in the use of plain language, with the greatest improvement occurring in the use of jargon (Wittenberg, Goldsmith, Ferrell, & Platt, 2015). Findings from this early work suggested providers might be able to change the way they communicate about medication and symptoms after receiving brief training.

Recognizing that provider pocket guides are a practical source of information and pocket guides for palliative care are valuable and useful (Critchley, Grantham, Plach, Bedard, & Oglan, 2002), we developed the PLP tool as a pocket guide for our national oncology nurse communication training program. The goal of this pilot study was to further determine whether PLP training would impact provider communication behavior by producing increased plain language when explaining a medication. To assess the use of plain language characteristics, we used role-play, a common form of communication skills training regardless of the level of training and experience of the participants. Role-play activities facilitate communication skill learning and practice by producing situations that involve self-disclosure, trust, respect, truth-telling, honesty, and reflective thinking (Babatsikou & Gerogianna, 2012).

## METHOD

A member of the research team facilitated an educational session on plain language, featuring the PLP pocket guide and a role-play activity, as part of a 2-day pain resource nurse-training course. Prior to and immediately after the session, participants (n = 87) were given a brief case description and asked to work in pairs to role-play a nurse-patient scenario. With random assignment to a medication-symptom pairing (bisacodyl for constipation, metoclopramide for nausea, and lorazepam for anxiety and worry), participants were asked to role-play the nurse’s explanation of the medication and how it would treat the symptom. These three pairings were selected as common symptoms in palliative care with commonly used medications and also included both physical and psychological symptoms.

One participant played the role of the nurse, and the other participant played the part of the patient. Before the role-play, both partners individually rated the nurse’s use of plain language characteristics. After the education session, participants were given a PLP pocket guide, presented with a different case and medication-symptom pair to role-play, and asked to switch roles so each participant had the opportunity to play the role of the nurse. This educational activity was determined to be exempt under the institutional review board at the supporting institution due to the educational scope of the exercise and study.

**Use of Plain Language**

Participants utilized a rating form based on the INDEX health literacy work of Kaphingst et al. (2012), which identifies best practices in improving health literacy demands. Plain language characteristics in the INDEX include the use of personal pronouns to recognize the patient/family, employ of active voice (e.g., "when you take this, drink plenty of water," vs. "when the medicine is taken, it requires plenty of water")/specific actions to demonstrate involvement with patient, explanation of the effect of the drug on the symptom to support patient/family delivery of care/medication, limited jargon to increase understanding, and limited length of spoken utterances to present information in small units to allow for feedback. Additional items based on a previous PLP study (Wittenberg et al., 2015) were utilized in the rating form as well and included disease/medication description, brand/generic explanation, use of patient name to demonstrate patient-centeredness, and use of the pocket card during the role-play. All rated items were specifically included as "plain language essentials" in the lecture participants received and were identified on the PLP pocket card. Participants were asked to rate the extent to which the nurse used plain language characteristics. Ratings were provided on a scale of 0 = never to 10 = a great deal.

**Statistical Analysis**

Demographics and rating items were summarized using the Statistical Package for the Social Sciences (SPSS) to produce descriptive statistics (frequency and mean scores).

## RESULTS

Overall, the sample had an average of 10 years of nursing experience. A total of 167 rating forms (80 before, 87 after) were collected from 87 participants. Participants in the session were predominantly Bachelor’s prepared nurses, working in an acute care hospital setting, with oncology as their clinical area of practice. Table 1 displays demographic characteristics of participants. The educational session featuring the PLP had excellent evaluation scores based on (1) not valuable to (5) very valuable scale: clarity of presentation (4.8), content of quality (4.7), and value to you as a clinician (4.7). A comparison between before and after responses (Table 2) showed improvement in the use of all plain language characteristics. The largest improvement in plain language skills was the explanation of brand and generic names, which rose from an average of 4.05 to an average of 7.5.

**Table 1:**
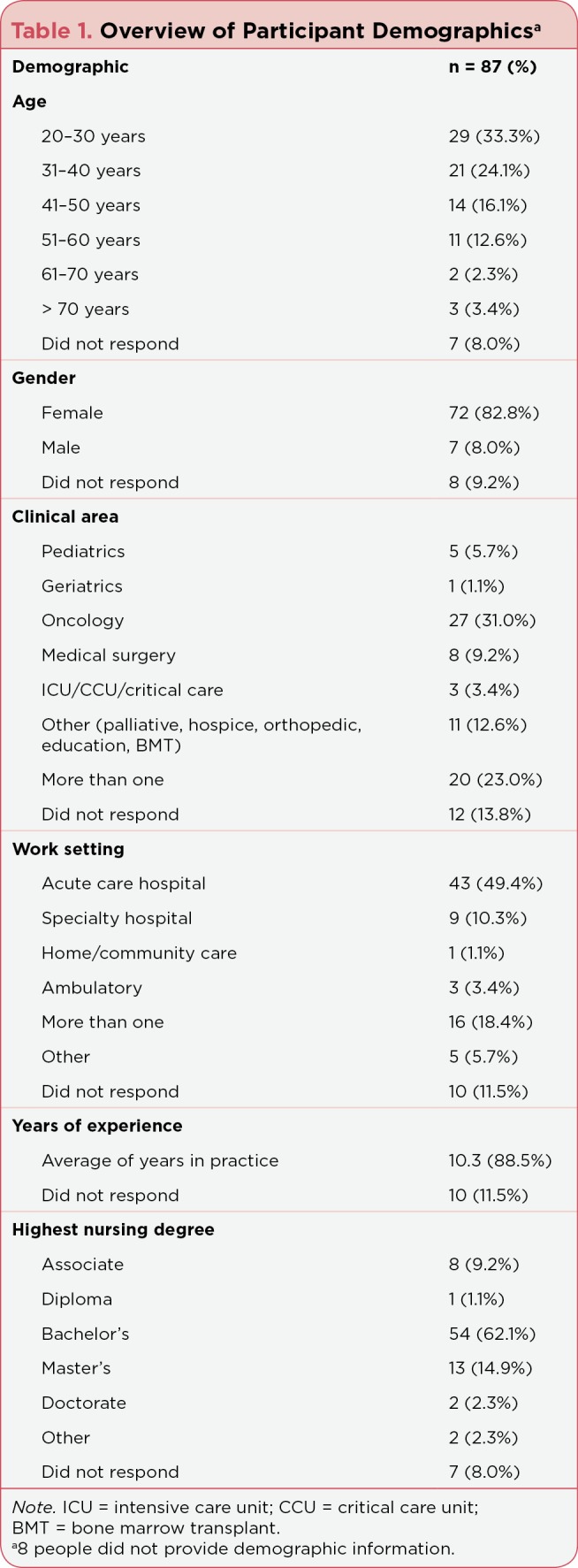
Overview of Participant Demographics^a^

**Table 2:**
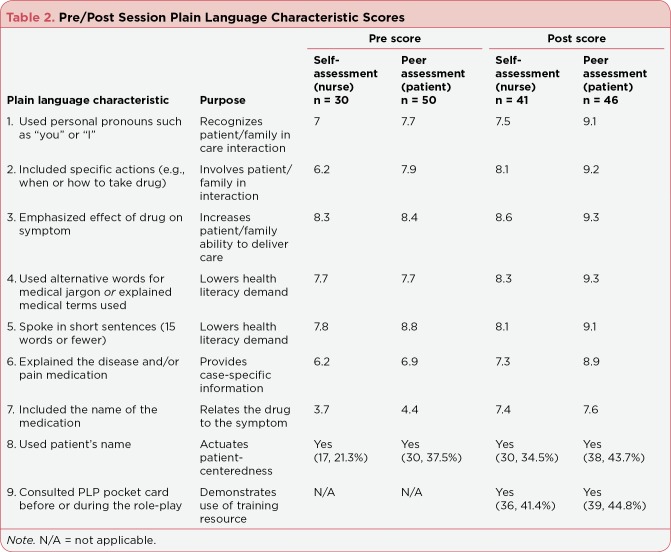
Pre/Post Session Plain Language Characteristic Scores

Across all characteristics (with exception of PLP pocket card use, as this was only rated after training), self and peer assessment scores improved after training (see Table 2). Greatest self-assessment gains were seen in describing specific actions (pre, 6.2/post, 8.1) and including the name of the medication in the role-play (pre, 3.7/post, 7.4). Peer assessment increase after the educational session was highest for including the name of the medication (pre, 4.4/post, 7.6) followed by explaining the disease and medication (pre, 6.9/post, 8.9). 

Both self and peer assessments showed the least improvement in reducing sentence length. The second lowest gain for both self and peer assessment was including the effect of a medication on a symptom. In post-educational session role-play, 86% of participants reported consulting the PLP pocket guide before or during the role-play. Overall, self-assessment ratings before and after training were consistently lower than peer assessment scores. 

## DISCUSSION AND CONCLUSION

Plain language is increasingly important as patient loads increase, varied populations seek care, and family caregivers face more responsibility in navigating symptom and pain management (Wittenberg-Lyles, Goldsmith, & Ferrell, 2013). Training health-care professionals to use spoken and written plain language to impact health literacy is relatively new. Ongoing provider training in plain language skills is needed, as health-care professionals continue to use terms potentially unfamiliar and confusing to patients (Bourquin, Stiefel, Mast, Bonvin, & Berney, 2015). The PLP pocket guide enables health-care providers to think about plain language as more than simply reducing clinical jargon. Providers need to focus on tailoring their communication to meet patient and family health literacy needs (Nouri & Rudd, 2015).

For nurses, medication management includes organization (acquiring, storing, tracking, and discarding medications), symptom knowledge, medication knowledge, and understanding and responding to the patient’s/family’s needs. The PLP pocket guide and training provide a communication strategy to help providers meet these needs in a clear and concise manner. Results of this study show the PLP as a promising way to improve providers’ plain language, enabling patient-centered communication when explaining medication. The training was accomplished in a short time span and can be integrated into any health-care professional program. Future research should test the PLP across patient populations (free downloadable use of the PLP can be accessed at communicatecomfort.com).

Nurses rated their own use of plain language lower than nurses rated peer usage. Although overall scores were improved, self-assessment scores suggest nurses thought they were least able to explain disease pathology and medication names. In similar research examining nurse communication with diabetic patients, nurses commonly clarified and repeated health information using medical jargon (Al Sayah, Williams, Pederson, Majumdar, & Johnson, 2014). This study’s findings suggest the utility of training for clinical practice using plain language resources. The PLP may be a useful tool for providers seeking resources as they seek to communicate complex information about medication. With a report from the National Academies of Sciences, Engineering, and Medicine (2016) calling for improved provider-patient communication, the PLP answers this call by supplying providers with understandable information.

Participants attending this training already represent a subset of this provider group and their interests in communication, health literacy, and pain management. Their own investments in matters of patient-centered care likely informed their responsiveness to the exercise described here. Although this study found PLP training and use of the pocket guide impacted nurse plain language use, further research is needed to determine whether these skills will be useful to patients and their families. It has been noted that a uniform approach to teaching communication skills does not meet the variety of needs presented by differing health-care disciplines (Turner, Payne, & O’Brien, 2011); however, plain language use may be an effective communication practice for all providers that impacts patient and family health literacy.

More research is necessary to understand the utility of plain language across health-care disciplines. Although self-assessment of communication has been shown to increase skills and learning, it should be noted that trainee self-assessments do not predict the quality of communication perceived by patients, family, or other evaluators (Dickson, Engelberg, Back, Ford, & Curtis, 2012). Future efforts to improve oncology providers’ use of plain language should address culture and English as a second language, both health literacy demands among growing patient populations. Further integration of the teach-back method with tools such as the PLP may offer further effective communication strategies. 

Patient and family health literacy includes the ability to speak with, understand, and process information shared by providers (Parnell, 2015); evidence has shown that lower health literacy is associated with poorer patient outcomes (National Academies of Sciences, Engineering, and Medicine, 2016). Providers are partially responsible for the literacy demands created in clinical learning situations with patients and families (Nouri & Rudd, 2015). Patient and family caregiver understanding of commonly used oncology terms is low, with most individuals misunderstanding 8 out of 10 terms, impacting their ability to participate in clinical consultations and activate recommended care (Pieterse, Jager, Smets, & Henselmans, 2013). The PLP pocket guide is one way oncology health-care providers can tailor communication to patient and family literacy needs, which may positively influence their participation. A copy of the PLP pocket guide is available for free as a download from the COMFORT Communication Project website (communicatecomfort.com).

**Acknowledgments**

Research reported in this publication was partially supported by the National Cancer Institute under award number R25CA174627. The content is solely the responsibility of the authors and does not necessarily represent the official views of the National Institutes of Health. 

## References

[1] Al Sayah F, Williams B, Pederson J L, Majumdar S R, Johnson J A (2014). Health literacy and nurses’ communication with type 2 diabetes patients in primary care settings.. *Nursing Research*.

[2] Babatsikou P F, Gerogianna K G (2012). The importance of role-play in nursing practice.. *Health Science Journal*.

[3] Badarudeen Sameer, Sabharwal Sanjeev (2010). Assessing readability of patient education materials: current role in orthopaedics.. *Clinical orthopaedics and related research*.

[4] Bourquin Céline, Stiefel Friedrich, Mast Marianne Schmid, Bonvin Raphael, Berney Alexandre (2015). Well, you have hepatic metastases: Use of technical language by medical students in simulated patient interviews.. *Patient education and counseling*.

[5] Centers for Disease Control and Prevention. (2016). Plain language materials & resources.. http://www.cdc.gov/healthliteracy/developmaterials/plainlanguage.html.

[6] Chou S W-y, Gaysynsky A, Persoskie A, Wittenberg E (2015). Health literacy and communication in palliative care.. *Textbook of Palliative Care Communication.*.

[7] Critchley Patrick P, Grantham Monica, Plach Nadia, Bedard Michel, Oglan Gerald (2002). An evaluation of the use of and satisfaction with the Palliative Care Pain and Symptom Pocket Card.. *Journal of palliative care*.

[8] Dickson Robert P, Engelberg Ruth A, Back Anthony L, Ford Dee W, Curtis J Randall (2012). Internal medicine trainee self-assessments of end-of-life communication skills do not predict assessments of patients, families, or clinician-evaluators.. *Journal of palliative medicine*.

[9] Hayes K, Gordon D B (2015). Delivering quality pain management: The challenge for nurses.. *Association of PeriOperative Registered Nurses Journal*.

[10] Howard Tera, Jacobson Kara L, Kripalani Sunil (2013). Doctor talk: physicians' use of clear verbal communication.. *Journal of health communication*.

[11] Kaphingst Kimberly A, Kreuter Matthew W, Casey Chris, Leme Luisa, Thompson Tess, Cheng Meng-Ru, Jacobsen Heather, Sterling Ryan, Oguntimein Joy, Filler Carl, Culbert Arthur, Rooney Megan, Lapka Christy (2012). Health Literacy INDEX: development, reliability, and validity of a new tool for evaluating the health literacy demands of health information materials.. *Journal of health communication*.

[12] National Academies of Sciences, Engineering, and Medicine. (2016). *Health literacy and palliative care: Workshop summary.*.

[13] Nouri Sarah S, Rudd Rima E (2015). Health literacy in the "oral exchange": an important element of patient-provider communication.. *Patient education and counseling*.

[14] Parnell T ((2015). *Health literacy in nursing: Providing person-centered care.*.

[15] Pieterse Arwen H, Jager Nienke A, Smets Ellen M A, Henselmans Inge (2013). Lay understanding of common medical terminology in oncology.. *Psycho-oncology*.

[16] Smith Meredith Y, DuHamel Katherine N, Egert Jennifer, Winkel Gary (2010). Impact of a brief intervention on patient communication and barriers to pain management: results from a randomized controlled trial.. *Patient education and counseling*.

[17] Turner Mary, Payne Sheila, O’Brien Terri (2011). Mandatory communication skills training for cancer and palliative care staff: does one size fit all?. *European journal of oncology nursing : the official journal of European Oncology Nursing Society*.

[18] Wittenberg Elaine, Goldsmith Joy, Ferrell Betty, Platt Christine Small (2015). Enhancing Communication Related to Symptom Management Through Plain Language.. *Journal of pain and symptom management*.

[19] Wittenberg-Lyles Elaine, Goldsmith Joy, Ferrell Betty (2013). Oncology nurse communication barriers to patient-centered care.. *Clinical journal of oncology nursing*.

[20] Wittenberg-Lyles E, Goldsmith J, Ferrell B, Ragan S (2012). *Communication in palliative nursing.*.

